# Simultaneous Detection and Differentiation of Four *Eimeria* Species in Chickens (*E. tenella*, *E. maxima*, *E. necatrix*, and *E. acervulina*) Using a Multiplex TaqMan-MGB qPCR Assay

**DOI:** 10.3390/ani15192792

**Published:** 2025-09-25

**Authors:** Lin Lin, Xiao-Li Chen, Sheng-Hui Wu, Xi Cai, Bin Jiang, Wei You, Min Zheng

**Affiliations:** 1 Institute of Animal Husbandry and Veterinary Medicine, Fujian Academy of Agricultural Sciences, Fuzhou 350013, China; linlin@faas.cn (L.L.); wush01@163.com (S.-H.W.); cici95743@163.com (X.C.); jiangbin83429588@163.com (B.J.); 17805951039@163.com (W.Y.); 2 Agricultural and Rural Bureau, Sanming 365000, China; chenxiaoli8212@163.com

**Keywords:** coccidiosis, *E. tenella*, *E. maxima*, *E. necatrix*, *E. acervuline*, multiplex real-time PCR, TaqMan-MGB

## Abstract

Chicken coccidiosis is a major parasitic disease in poultry that causes substantial economic losses worldwide. The accurate and simultaneous diagnosis of mixed infections with multiple *Eimeria* species remains challenging using conventional methods. In this study, we developed a multiplex TaqMan-MGB qPCR assay targeting the ITS1 region for simultaneous detection and quantification of four major pathogenic species: *E. acervulina*, *E. necatrix*, *E. maxima*, and *E. tenella.* The assay exhibited high specificity without cross-reactivity and achieved a sensitivity 100-fold greater than conventional PCR, with detection limits ranging from 10^1^ to 10^2^ copies/μL. Standard curves demonstrated strong linearity (R^2^ ≥ 0.95) and amplification efficiency (95–113%). Repeatability was robust, with intra- and inter-assay CVs below 2%. Applied to 165 clinical samples collected from poultry farms in Fujian Province, China (2022–2024), the assay detected an overall *Eimeria* infection rate of 93.3%, higher than conventional PCR (89.7%). Epidemiological analysis revealed *E. tenella* (78.8%, [130/165]) and *E. necatrix* (78.8%, [130/165]) were dominant, while mixed infections were frequent (83.3%, [136/154]), including dual (45.5%, [70/154]), triple (31.2%, [48/154]), and quadruple (11.7%, [18/154]) infections. This multiplex TaqMan-MGB qPCR provides a rapid, sensitive, and quantitative tool for detecting multiple *Eimeria* species, greatly improving diagnostic efficiency and supporting clinical diagnosis, surveillance, and control strategies.

## Simple Summary

Chicken coccidiosis, caused by *Eimeria* spp., is a major economic burden to the global poultry industry, yet the accurate diagnosis of mixed infections remains challenging. This study aimed to develop a highly sensitive and specific quadruplex TaqMan-MGB real-time PCR assay for the simultaneous detection of four key *Eimeria* species: *E. acervulina*, *E. necatrix*, *E. maxima*, and *E. tenella*. The assay demonstrates high sensitivity and specificity, with a detection limit as low as 10^1^ to 10^2^ DNA copies/μL. Validation with 165 suspected clinical samples from Fujian, China (2022–2024) revealed a 93.3% infection rate, higher than conventional PCR (89.7%). *E. tenella* (78.8%) and *E. necatrix* (62.4%) were most common, with mixed infections in 83.3% of cases (dual, triple, or quadruple species). This assay offers an efficient tool for early clinical diagnosis, large-scale epidemiological surveys, and targeted control strategies for coccidiosis in poultry production.

## 1. Introduction

Chicken coccidiosis, caused by *Eimeria* spp., remains one of the most economically significant parasitic diseases in the global poultry industry [1], leading to annual losses exceeding 14.5 billion USD worldwide [2,3]. Avian coccidiosis primarily affects the intestinal tract of chickens, causing diarrhea, dehydration, weight loss, lack of appetite and weakness, in severe cases, mortality [4]. It is estimated that coccidiosis is endemic in poultry farms globally, with billions of dollars spent annually on prevention and treatment, severely constraining the sustainable development of the poultry industry [5].

Currently, ten *Eimeria* species are recognized to infect chickens: *E. acervulina*, *E. maxima*, *E. tenella*, *E. necatrix*, *E. brunetti*, *E. mitis*, *E. praecox*, *E. lata*, *E. nagambie*, and *E. zaria* [6,7]. These parasites differ in pathogenicity and intestinal localization [8]. *E. acervulina* (duodenum) and *E. maxima* (mid-intestine) mainly cause malabsorption and subclinical losses, whereas *E. tenella* (caeca) and *E. necatrix* (mid-intestine) induce severe hemorrhagic lesions and high mortality [9,10,11]. Other species—including *E. brunetti* (ileum, rectum), *E. mitis* (ileum), and *E. praecox* (duodenum)—also occur; *E. mitis* and *E. praecox* are generally less pathogenic but may impair feed efficiency, especially in mixed infections. Newly described *E. lata*, *E. nagambie*, and *E. zaria* replicate in the duodenum and jejunum, underscoring the expanding diversity of avian coccidia [6]. Despite these differences, all species share a fecal–oral transmission route [12,13]. Among different species, *Eimeria tenella*, *Eimeria acervulina*, *Eimeria maxima*, and *Eimeria necatrix* are frequently considered to impose the greatest economic threat to chicken production worldwide, due to their combined pathogenicity, wide distribution, and substantial impact on productivity [4,9].

The prevalence of coccidiosis is ubiquitous in commercial poultry production systems. It ranks among the top three poultry diseases in the United Kingdom and is considered one of the ten most important veterinary challenges affecting resource-limited communities in South Asia [14,15]. Regional epidemiological studies have reported high infection rates: 100% in broiler farms in Algeria (commonly as mixed infections) [16], 98% in Australian commercial flocks [17], and 85.7% in Greek farms [18]. Similarly, high prevalence and complex mixed infections have been documented in backyard production systems in Argentina and Chile [3]. In major poultry-producing provinces of China, such as Shandong, Sichuan, and Hebei, the prevalence reaches 95%, with *E. acervulina*, *E. necatrix*, *E. tenella*, and *E. maxima* identified as the most prevalent species, and mixed infections being the norm under field conditions [19].

Coccidiosis exhibits a high global prevalence across diverse production systems, yet its accurate diagnosis remains challenging due to the limitations of conventional and early molecular methods. Traditional diagnosis of *Eimeria* species has relied on oocyst morphology, infection site, and histopathology [20], yet these methods remain laborious, time-consuming, and prone to misidentification—especially in mixed infections or among morphologically similar species [21,22]. While molecular techniques such as conventional PCR, multiplex PCR, RAPD, LAMP, SSCP and qPCR [23,24,25,26,27,28,29] have improved detection, many still lack the accuracy needed for discriminating genetically diverse or highly homologous strains in co-infections. Although conventional multiplex PCR techniques, enabled simultaneous detection of several *Eimeria* species [26], they frequently exhibited limitations in sensitivity, specificity, and quantitative capacity—particularly under high-template diversity or when discriminating highly homologous sequences.

TaqMan-based multiplex qPCR addresses these gaps by allowing efficient multi-target detection in a single reaction, reducing time, cost, and contamination risk [30]. The use of minor groove binder (MGB) probes further increases specificity and stability—enabling precise differentiation of closely related sequences and reliable parasite load quantification [31]. This approach is essential for accurate diagnosis of mixed infections, large-scale surveillance, and targeted control of coccidiosis.

Based on these considerations and given the high prevalence and pathogenicity of *E. tenella*, *E. necatrix*, *E. maxima*, and *E. acervulina* [4,9], this study aimed to establish a multiplex TaqMan-MGB real-time PCR assay for the simultaneous detection, quantification, and precise differentiation of these four majors pathogenic *Eimeria* species. The developed platform demonstrates high sensitivity, strong specificity, and reliable quantitative performance, offering crucial technical support for early clinical diagnosis, large-scale epidemiological surveillance, and targeted control of avian coccidiosis.

## 2. Materials and Methods

### 2.1. Parasite Strains and DNA Samples

Strains of *Eimeria tenella*, *Eimeria maxima*, *Eimeria necatrix*, and *Eimeria acervulina* were isolated and maintained by the Animal Diagnosis Center of Fujian Academy of Agricultural Sciences. As negative controls, DNA samples of *E. brunetti* and *E. mitis* were extracted from a commercially available bivalent live vaccine (containing attenuated strains of *Eimeria brunetti* PBBD and *Eimeria mitis* PMiBD) purchased from Foshan Zhengdian Biological Technology Co., Ltd. (Foshan, China).DNA samples from other parasites were obtained from our laboratory stock, including *Histomonas meleagridis*, *Trichomonas gallinae*, *Ascaridia galli*, *Capillaria* spp., *Tetrameres* spp., *Syngamus trachea*, *Heterakis gallinarum*, *Raillietina* spp., *Hymenolepis* spp., *Echinostoma* spp., and *Leucocytozoon* spp.

### 2.2. Clinical Samples

A total of 165 suspected *Eimeria*-infected samples (including 53 fecal samples and 112 intestinal specimens) were collected from clinical submissions to our veterinary diagnostic center between 2022 and 2024. These samples were submitted by either farm veterinarians or poultry farmers from commercial chicken farms across southeastern China for parasitological diagnosis. All samples were submitted with the informed consent of farm owners for diagnostic purposes. Subsequent use of the samples for research was conducted in accordance with institutional and ethical guidelines.

### 2.3. Genomic DNA Preparation

For fecal samples, approximately 200 mg of fresh feces was homogenized in 1 mL of sterile phosphate-buffered saline (PBS, pH 7.4) and filtered through double-layer sterile gauze to remove large debris. The filtrate was centrifuged at 3000× *g* for 10 min, and the pellet containing *Eimeria* oocysts was washed twice with PBS. For intestinal content samples, approximately 200 mg of material from the cecum, mid-intestine, or duodenum (depending on suspected site of infection) was collected and processed in the same manner. Total genomic DNA was extracted using a commercial stool DNA extraction kit (Mag-Bind^®^ Stool DNA Kit, omega BIO-TEK, Norcross, GA, USA), All DNA samples were subsequently used as templates for the multiplex TaqMan-MGB qPCR assay.

### 2.4. Primers and Probes for Multiplex qPCR

The ITS-1 gene sequences of *Eimeria acervulina* (GenBank accession: AF446055), *E. tenella* (AF446074), *E. maxima* (AF4460560), and *E. necatrix* (AY571579) were retrieved from the GenBank database as target sequences for primer and probe design. Species-specific primers and TaqMan-MGB probes were designed using Primer Premier 5.0 software, ensuring intra-species conservation and inter-species specificity. The specificity of all primers and probes was validated using the BLAST tool (2.17.0, NCBI, Bethesda, MD, USA) to exclude cross-reactivity with non-target sequences. Potential secondary structure s (e.g., primer-dimers and hairpins) were analyzed and minimized using the PrimerSelect module in DNASTAR Lasergene 17.6 software (DNASTAR, Madison, WI, USA). Multiple primer-probe combinations were initially designed and experimentally screened to identify the optimal set with the highest amplification efficiency. The TaqMan-MGB probes were 5′-labeled with distinct fluorophores: FAM for *E. acervulina.* VIC for *E. maxima*, Texas Red for *E. tenella*, and Cy5 for *E. necatrix.* All probes were modified with a 3′ minor groove binder (MGB) to enhance binding specificity and increase melting temperature. The sequences of primers and probes are listed in Table 1 and were synthesized by Sangon Biotech (Shanghai, China).

### 2.5. Construction of Standard Plasmids

Species-specific primer pairs targeting the ITS-1 gene regions of *E. acervulina*, *E. maxima*, *E. tenella*, and *E. necatrix* (primer sequences listed in Table 2) were designed to amplify fragments containing the TaqMan-qPCR target sequences. Each PCR was performed in a 20 μL reaction mixture containing 10 μL of 2× Taq Master Mix (Dye Plus) (Vazyme Biotech Co., Ltd., Nanjing, China), 2 μL of DNA template, 1 μL of each primer (10 μM), and 6 μL of nuclease-free water (ddH_2_O). The cycling conditions were as follows: initial denaturation at 95 °C for 3 min, followed by 35 cycles of 95 °C for 15 s, 58 °C for 15 s, and 72 °C for 30 s, with a final extension at 72 °C for 5 min.

The PCR products were purified and ligated into the pMD18-T vector (Takara Bio Inc., Shiga, Japan) to generate the recombinant plasmids *p*-acer, *p*-maxi, *p*-ten, and *p*-nec. The correctness of the recombinant constructs was confirmed by Sanger sequencing. Plasmid concentration and purity were determined using a DS-11 spectrophotometer (DeNovix Inc., Wilmington, DE, USA). The copy number of each recombinant plasmid was calculated using the following formula: Copies/μL = (6.02 × 10^23^) × (X ng/μL × 10^−9^)/(plasmid length (bp) × 660), where X represents the plasmid concentration in ng/μL.

### 2.6. Establishment of the Multiplex TaqMan qPCR Reaction System

A matrix approach was employed to determine the optimal concentrations of primers and probes. Both primer and probe concentrations were optimized separately to achieve the best reaction conditions. Primers and probes were diluted to final concentrations ranging from 0.1 to 0.6 μmol/mL and subjected to gradient amplification. Reaction conditions for real-time TaqMan qPCR were optimized by testing different system compositions and various combinations of primer and probe concentrations, as well as annealing temperatures. The optimal reaction system, primer and probe concentrations, and annealing temperature were then selected to establish the multiplex TaqMan qPCR assay.

### 2.7. Standard Curve Creation

The recombinant plasmid standards *p*-acer, *p*-max, *p*-ten, and *p*-nec were serially diluted 10-fold (from 10^8^ to 10^4^ copies/μL). Each dilution was tested in triplicate using the TaqMan qPCR assay. The mean quantification cycle (Cq) value from these three technical replicates was then used to generate the standard curve and the corresponding linear regression equation for each *Eimeria* species. The logarithm of the initial template copy number was plotted on the x-axis, and the corresponding Cq values were plotted on the y-axis to generate regression curves. Standard curves were separately constructed for *E. acervulina*, *E. maxima*, *E. tenella*, and *E. necatrix*, establishing the quantitative relationship between plasmid copy number and Cq value for each target species.

### 2.8. Sensitivity, Specificity, and Reproducibility Evaluations

To assess assay specificity, DNA from other parasites, including: *Eimeria brunetti*, *Eimeria mitis*, *Histomonas meleagridis*, *Trichomonas gallinae*, *Ascaridia galli*, *Capillaria* spp., *Tetrameres* spp., *Syngamus trachea*, *Heterakis gallinarum*, *Raillietina* spp., *Hymenolepis* spp., *Echinostoma* spp., and *Leucocytozoon* spp. were used as templates. Fecal samples from healthy chickens confirmed negativity for *Eimeria* spp. by microscopy and PCR and were used as negative controls. Specificity was evaluated by performing the multiplex TaqMan-MGB qPCR assay with these samples.

To further assess the sensitivity of the assay, the constructed plasmid standards *p*-acer, *p*-max, *p*-ten, and *p*-nec were subjected to 10-fold serial dilutions until no amplification signal was detected by the real-time PCR system. The lowest detectable genomic copy number for each target was thereby determined, representing the assay’s limit of detection (LOD). The sensitivity of the developed TaqMan qPCR assay for each *Eimeria* species was compared with that of conventional PCR, the standard molecular method for *Eimeria* detection, to validate its improved sensitivity and specificity.

In addition, the established TaqMan qPCR assay was further evaluated for repeatability by performing three independent runs on different days using the four positive quality control plasmids as templates. Intra-assay and inter-assay variations were calculated based on the Cq values, and the coefficient of variation (CV) was calculated using the formula: CV (%) = (Standard deviation (SD)/Mean (X)) × 100 to evaluate the assay’s reproducibility and stability.

### 2.9. Evaluation of Clinical Samples by Quadruplex TaqMan Real-Time PCR and Conventional PCR

The established quadruplex TaqMan real-time PCR assay was applied to detect 165 clinical samples (including feces and intestinal tissues) suspected of *Eimeria* infection, which were submitted from various poultry farms in Fujian Province, China, between 2022 and 2024. In parallel, all samples were also tested using a conventional PCR assay, and the concordance rate between the two methods was calculated.

### 2.10. Data and Statistical Analysis

qPCR Data Processing: Raw Cq values were determined using automatic baseline settings and a manually set threshold within the qPCR instrument software (LightCycler^®^ 96, Roche, Basel, Switzerland). All samples and standard curves were run in triplicate, and mean Cq values were used for subsequent analysis.

Standard Curve and Efficiency: Standard curves were generated for each target by plotting the mean Cq value against the logarithm of the known template copy number. Amplification efficiency (E) for each assay was calculated automatically by the instrument’s built-in software based on the slope of the standard curve.

Specificity and Sensitivity: Analytical specificity was determined by testing against a panel of non-target parasites. The limit of detection (LOD) was defined as the lowest copy number per reaction detectable in ≥95% of replicates.

Reproducibility: Intra-assay variability was assessed from the standard deviation of Cq values across triplicate wells within a single run. Inter-assay variability was determined from replicates across three independent runs. Both are expressed as coefficients of variation (CV, %).

Clinical Sample Analysis: A sample was considered positive if amplification occurred at a Cq value < 40. Prevalence and co-infection rates were calculated as percentages with 95% confidence intervals where appropriate.

## 3. Results

### 3.1. Establishment and Optimization of the Multiplex Real-Time TaqMan-MGB PCR Assay

Following systematic optimization, the final multiplex TaqMan-MGB real-time PCR assay was established in a total reaction volume of 20 μL, containing 10 μL of 2× All-Powerful qPCR PreMix (Vazyme Biotech, Nanjing, China), 0.4 μL each of forward and reverse primers (final concentration: 0.2 μM for each primer), 0.2 μL each of the corresponding TaqMan-MGB probes (final concentration: 0.1 μM for each probe), and 2 μL of mixed template DNA, with nuclease-free water added to reach the final volume. The optimized thermal cycling conditions were as follows: uracil-N-glycosylase digestion at 53 °C for 10 min, initial denaturation at 95 °C for 30 s, followed by 45 cycles of denaturation at 95 °C for 10 s and annealing/extension at 60 °C for 20 s. These optimized conditions enabled efficient amplification and high specificity for the simultaneous detection of the four *Eimeria* species in chickens.

### 3.2. Standard Curve Preparation and Evaluation

**Figure 1:**
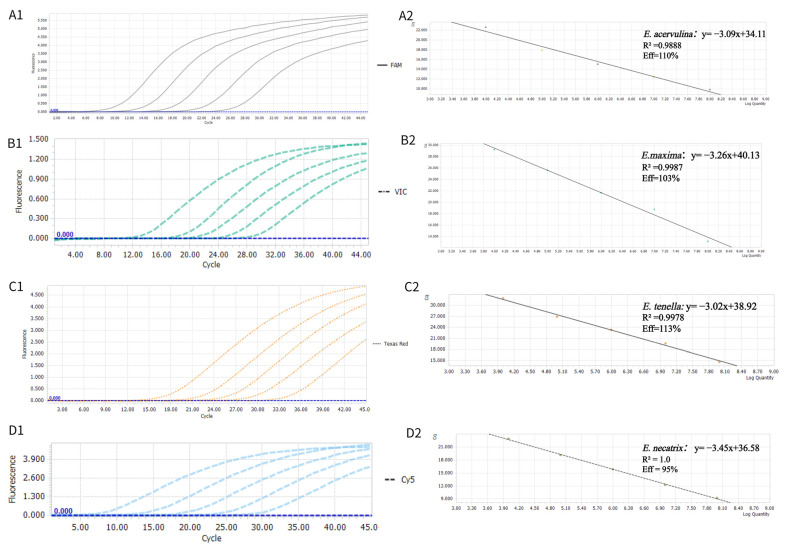
The amplification curves and standard curves of the TaqMan-MGB qPCR assay. The concentrations ranging from 10^8^ to 10^4^ copies/µL. (**A1**,**A2**), (**B1**,**B2**), (**C1**,**C2**) and (**D1**,**D2**) are the amplification and Standard curves of the standard plasmids of *p*-acer, *p*-max, *p*-ten, and *p*-nec, respectively.

The constructed standard plasmid solutions were serially diluted tenfold (10^8^–10^4^ copies/μL) with DNase/RNase-free water and used as templates for the multiplex TaqMan-MGB real-time PCR assay. A standard curve was generated by plotting the logarithm of the initial plasmid genome copy number (x-axis) against the Cq values (y-axis) (Figure 1). The linear regression equations and performance parameters were as follows: *E. acervulina*: y = −3.09x + 34.11, amplification efficiency 110%, R^2^ = 0.988; *E. maxima*: y = −3.259x + 40.13, amplification efficiency 102%, R^2^ = 0.9987; *E. necatrix*: y = −3.45x + 36.58, amplification efficiency 95%, R^2^ = 1.000; and *E. tenella*: y = −3.02x + 38.92, amplification efficiency 113%, R^2^ = 0.9978. These results indicate that the established multiplex real-time PCR system exhibits excellent linear correlation and optimal detection performance across different concentrations of target nucleic acids, and the initial gene copy number of *Eimeria* nucleic acids in test samples can be calculated by substituting the corresponding Cq values into the regression equations.

### 3.3. Specificity Evaluation

**Figure 2:**
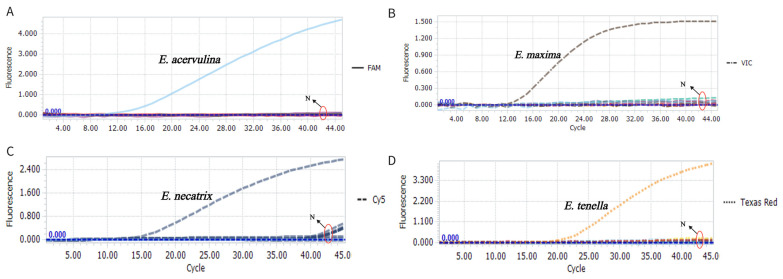
The specificity of the multiplex TaqMan-MGB qPCR assay. Amplification plots are shown for (**A**) *E. acervulina* (FAM channel), (**B**) *E. maxima* (VIC channel), (**C**) *E. necatrix* (Cy5 channel), (**D**) *E. tenella* (Texas Red channel). N means controls used in this study, including *E. brunetti*, *E. mitis*, *Histomonas meleagridis*, *Trichomonas gallinae*, *Ascaridia galli*, *Capillaria* spp., *Tetrameres* spp., *Syngamus trachea*, *Heterakis gallinarum*, *Raillietina* spp., *Hymenolepis* spp., *Echinostoma* spp., and *Leucocytozoon* spp. No positive signals were detected in the controls.

To evaluate the specificity of the multiplex TaqMan-MGB qPCR assay and assess potential cross-reactivity, DNA from *E. brunetti*, *E. mitis*, *E. praecox* and other common chicken parasites (*Histomonas meleagridis*, *Trichomonas gallinae*, *Ascaridia galli*, *Capillaria* spp., *Tetrameres* spp., *Syngamus trachea*, *Heterakis gallinarum*, *Raillietina* spp., *Hymenolepis* spp., *Echinostoma* spp., and *Leucocytozoon* spp.) was tested. Plasmids containing target sequences for *E. acervulina*, *E. maxima*, *E. tenella*, and *E. necatrix* served as positive controls, and fecal samples from healthy chickens were used as negative controls. Fluorescence signals were detected exclusively in the corresponding channels *(E. acervulina* in FAM, *E. maxima* in VIC, *E. tenella* in Texas Red, *E. necatrix* in Cy5), with no amplification from the other 15 parasite species or negative controls (Figure 2), confirming the assay’s high specificity and absence of cross-reactivity for the four target *Eimeria* species.

### 3.4. Sensitivity Evaluation

**Figure 3:**
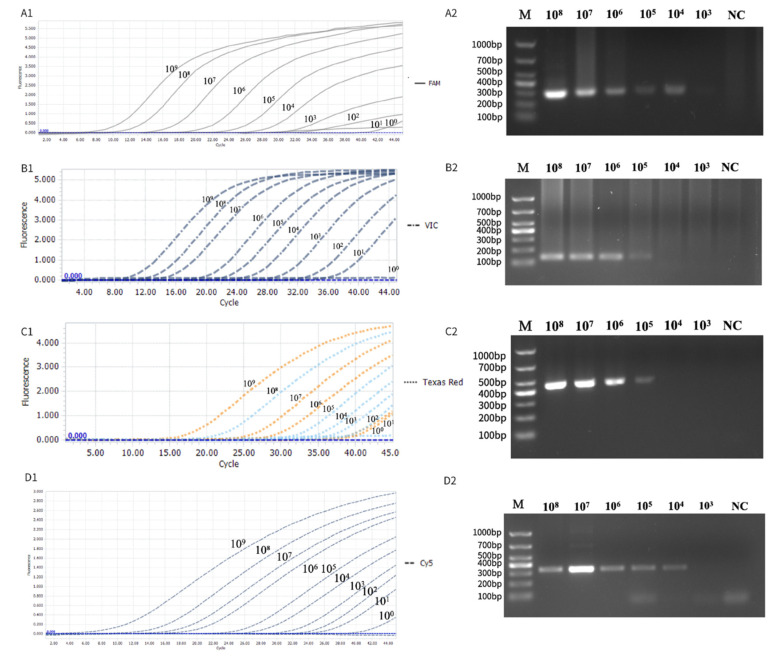
Sensitivity comparison between multiplex TaqMan-MGB qPCR and conventional PCR for detecting *Eimeria acervulina* (**A1**,**A2**), *Eimeria maxima* (**B1**,**B2**), *Eimeria tenella* (**C1**,**C2**), and *Eimeria necatrix* (**D1**,**D2**). Panels (**A1**–**D1**) show real-time fluorescence amplification curves generated from 10-fold serial dilutions (10^9^ to 10^0^ copies/μL) of standard plasmids in the FAM (**A1**), VIC (**B1**), Texas Red (**C1**), and Cy5 (**D1**) channels, respectively. Panels (**A2**–**D2**) show corresponding agarose gel electrophoresis results of conventional PCR products from the same dilution series. M: DNA marker; NC: negative control.

To determine the sensitivity of the developed multiplex TaqMan-MGB real-time PCR assay, standard plasmids *p*-acer, *p*-max, *p*-ten, and *p*-nec were subjected to 10-fold serial dilutions ranging from 10^9^ to 10^0^ copies/μL and used as templates for detection. Conventional PCR was performed in parallel for comparison. The multiplex TaqMan-MGB qPCR assay achieved detection limits of 10^2^ copies/μL for p-acer (*E. acervulina*), compared to 10^4^ copies/μL by conventional PCR; 10^1^ copies/μL for p-max (*E. maxima*), compared to 10^5^ copies/μL by conventional PCR; 10^2^ copies/μL for p-ten (*E. tenella*), compared to 10^5^ copies/μL by conventional PCR; and 10^1^ copies/μL for p-nec (*E. necatrix*), compared to 10^4^ copies/μL by conventional PCR (Figure 3). These results demonstrate that the sensitivity of the multiplex TaqMan-MGB qPCR assay is markedly higher than that of conventional PCR for all four target *Eimeria* species.

According to the established interpretation criteria, samples with Cq values < 40 and exhibiting a typical S-shaped amplification curve were considered positive. Samples with Cq values > 40 or without detectable Cq values and lacking a characteristic amplification curve were considered negative.

### 3.5. Reproducibility of the Multiplex TaqMan-MGB qPCR Assay

To assess the reproducibility of the multiplex TaqMan-MGB real-time PCR assay, standard plasmids p-acer, p-max, p-ten, and p-nec were tested at three different dilution levels (10^7^ to 10^5^ copies/μL) to determine intra-assay and inter-assay variability. The coefficient of variation (CV) of Cq values was calculated for each replicate to evaluate assay repeatability. As shown in Table 3, the intra-assay CVs for p-acer, p-max, p-ten, and p-nec ranged from 0.76% to 1.86%, while inter-assay CVs ranged from 0.91% to 1.88%. All CV values were below 2%, indicating that the developed multiplex qPCR assay demonstrated excellent reproducibility and stability across different template concentrations.

### 3.6. Detection of Clinical Samples

**Figure 4:**
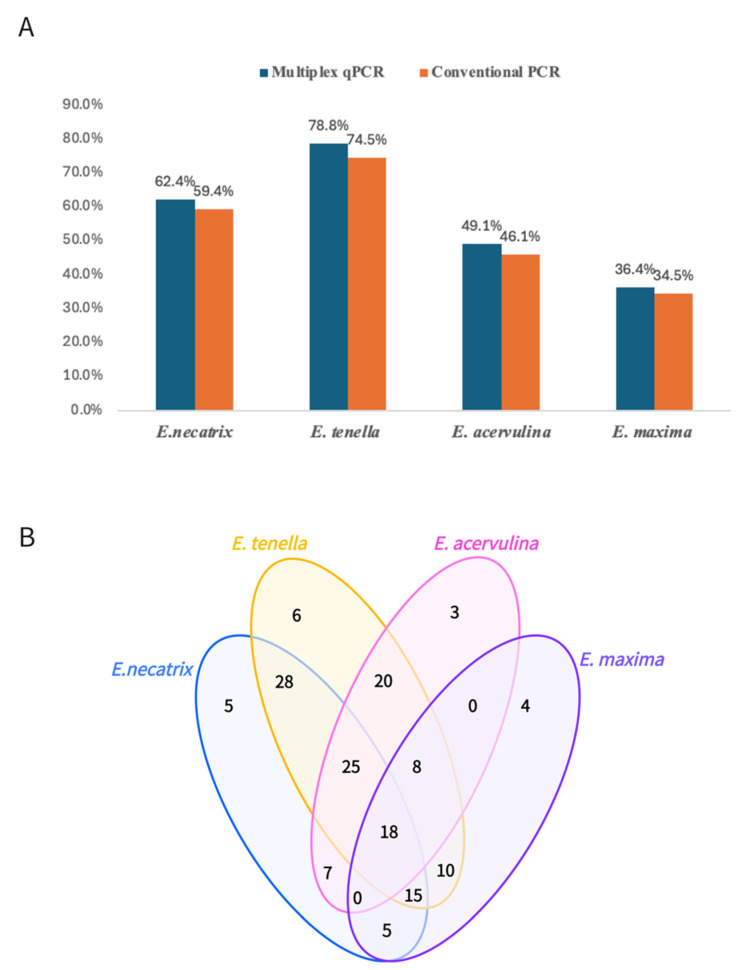
(**A**) Comparison of detection rates for *Eimeria acervulina*, *E. tenella*, *E. maxima*, and *E. necatrix* in 165 clinical samples using the multiplex TaqMan-MGB qPCR assay (blue bars) and conventional PCR (orange bars). The multiplex qPCR method demonstrated higher detection rates for all four target species compared to conventional PCR. (**B**) Venn diagram illustrating the distribution of single and mixed infections among the four *Eimeria* species detected in 154 qPCR-positive clinical samples. Mixed infections were predominant, with the most frequent combinations being *E. tenella* + *E. necatrix*, and *E. acervulina* + *E. tenella* + *E. necatrix*. Four-species co-infection was observed in 18 samples.

Using the multiplex TaqMan-MGB qPCR assay developed in this study, a total of 165 suspected *Eimeria*-infected samples, comprising 53 fecal samples and 112 intestinal specimens, were collected from clinical submissions to our veterinary diagnostic center between 2022 and 2024 and tested in parallel with conventional PCR for comparison. The overall detection rate of the multiplex TaqMan-MGB qPCR assay for the four target *Eimeria* species was 93.3% (154/165), which was higher than that of conventional PCR (89.7%, [148/165]). The detection rates for each species were also higher than those obtained by the traditional method: *Eimeria tenella* (78.8% [130/165] vs. 74.5% [123/165]), *Eimeria necatrix* (62.4% [103/165] vs. 59.4% [98/165]), *Eimeria acervuline* (49.1% [81/165] vs. 46.1% [76/165]), and *Eimeria maxima* (36.4% [60/165] vs. 34.5% [57/165]) (Figure 4A). Among the four species, *E. tenella* exhibited the highest prevalence (78.8% [130/165]), followed by *E. necatrix* (62.4% [103/165]), while *E. maxima* had the lowest prevalence (36.4% [60/165]). Mixed infections were highly prevalent, with an overall co-infection rate of 83.3% (136/154) among the positive samples. Double infections accounted for 45.5% (70/154) of positives, with *E. acervulina* + *E. tenella* and *E. tenella* + *E. necatrix* being the most frequent combinations. Triple infections represented 31.2% (48/154) of positives, most commonly *E. acervulina* + *E. tenella* + *E. necatrix*. Quadruple infections were detected in 11.7% (18/154) of positive cases. The results demonstrate that the multiplex TaqMan-MGB qPCR assay exhibits superior sensitivity compared to conventional PCR while reliably identifying mixed infections and simultaneously detecting four *Eimeria* species in a single reaction, significantly enhancing both clinical diagnostic efficiency and large-scale epidemiological surveillance capabilities.

## 4. Discussion

Coccidiosis remains one of the most economically devastating parasitic diseases in the poultry industry, causing reduced feed conversion, stunted growth, morbidity, and high mortality in severe cases [32]. The complex life cycle of *Eimeria* spp. involves both asexual and sexual stages in intestinal epithelial cells, leading to significant tissue damage [33]. Infections occur through ingestion of sporulated oocysts from contaminated feed, water, or the environment, with the resistant oocyst wall enabling survival for months [34]. Once ingested, sporozoites invade epithelial cells and undergo schizogony, gametogony, and oocyst formation, perpetuating the infection cycle [35]. A single oocyst can yield up to 800,000 progenies, rapidly contaminating the environment and complicating prevention [11].

Epidemiological studies confirm that mixed-species and subclinical infections are widespread globally and represent a dominant pattern in poultry production [36]. Surveys in Guangdong, Hubei, Anhui, and Jiangsu reported mixed infection rates of 65–100%, mainly involving *E. acervulina*, *E. tenella*, *E. maxima*, and *E. necatrix* [37,38,39]. Similar prevalence has been recorded in Australia, Korea, and Colombia (54.3–100%), with up to 5–7 species detected [17,40,41]. Such co-infections may exacerbate disease severity through synergistic interactions [10], underscoring the importance of early and accurate diagnosis.

Although single plex qPCR and some multiplex PCR assays for individual *Eimeria* species have been reported, multiplex systems capable of detecting several species in a single reaction are rare [26,42]. TaqMan-based multiplex qPCR offers clear advantages, including simultaneous detection of multiple targets, reduced time and cost, lower contamination risk, and quantitative capability. Quantification of parasite loads is especially valuable for evaluating mixed infections and guiding control strategies.

The ITS1 region of ribosomal DNA is an ideal molecular target due to inter-species variability and intra-species conservation [43]. It has been used in detecting *Toxoplasma gondii*, *Cryptosporidium* spp., and multiple *Eimeria* species [26,44]. Its multi-copy nature also enhances detection sensitivity [45]. In this study, species-specific primers and TaqMan-MGB probes targeting ITS1 were designed, with four fluorophores and non-fluorescent quenchers reducing background signals and improving specificity. No cross-reactivity with other intestinal parasites was observed, confirming high analytical specificity.

Subclinical infections, though lacking clinical signs, can reduce performance and egg production [46,47]. Fadunsin et al. reported higher subclinical infection rates in chicks (52.7%) than in adult birds (21.1%), which are often missed by conventional diagnostics [48]. Our assay achieved detection limits of 10^1^–10^2^ copies/μL, over 100-fold more sensitive than conventional PCR, with intra- and inter-assay CVs < 2%. This enables reliable detection of asymptomatic carriers and strengthens surveillance. Although a sporulated oocyst theoretically provides eight genomic copies, field sensitivity may be affected by sample collection and DNA extraction losses.

Field application revealed a 93.3% prevalence of *Eimeria* in Fujian Province, with *E. tenella* (78.8%) and *E. necatrix* (62.4%) being dominant. Mixed infections (83.3%) were common, including dual (45.5%), triple (31.2%), and quadruple (11.7%) infections. The frequent *E. tenella* + *E. necatrix* and *E. acervulina* + *E. tenella* + *E. necatrix* combinations align with previous reports, likely due to oocyst resilience, incomplete disinfection, non-overlapping intestinal sites, and synergistic pathogenic effects [42,43]. Such co-infections can exacerbate intestinal damage, delay recovery, and impair drug or vaccine efficacy.

The subtropical climate of Fujian, with high temperature and humidity, may further facilitate oocyst sporulation and persistence, increasing infection risk through contaminated feed, water, and litter [4,20]. Thus, molecular surveillance combined with strengthened farm management is essential to control co-infections.

This study established a multiplex TaqMan-MGB qPCR assay targeting ITS1 that enables sensitive, specific, and simultaneous detection of four major *Eimeria* species. Compared with conventional diagnostics, it significantly improves accuracy, particularly for mixed and subclinical infections, and provides a reliable tool for early diagnosis and large-scale epidemiological surveillance, supporting more effective coccidiosis control strategies in poultry production.

## 5. Conclusions

In this study, we developed and validated a multiplex TaqMan-MGB real-time qPCR assay targeting the ITS1 region for the simultaneous detection and quantification of four major *Eimeria* species in chickens—*E. acervulina*, *E. maxima*, *E. necatrix*, and *E. tenella.* The assay demonstrated high analytical sensitivity (10^1^–10^2^ copies/μL), excellent specificity with no cross-reactivity, and strong reproducibility (CV < 2%). Field application revealed higher detection rates than conventional PCR and confirmed the predominance of mixed-species infections, underscoring the complex epidemiology of coccidiosis in poultry farms. This multiplex assay provides a rapid, accurate, and efficient diagnostic platform for clinical diagnosis and epidemiological surveillance. Future studies should extend its application to additional *Eimeria* species, validate its performance across diverse regions, and explore its role in monitoring anticoccidial drug efficacy and vaccine evaluation.

## Figures and Tables

**Table 1:** Primers and probes for multiplex qPCR detection of *E. acervulina*, *E. maxima*, *E. tenella*, and *E. necatrix*.

Primes	Sequence (5′ → 3′)	Target Gene	Product Size (bp)
*acer*-F	AAGCATCATTGCCACCT	*Eimeria acervulina* AF446055 ITS-1	140
*acer*-R	TGCCAGGGTCACATGT		
*acer*-probe	*FAM*-CGGCGCATGCACCGCT-*MGB*		
*max*-F	ATCATTGAATCCCTTTCA	*Eimeria maxima* AF446060 ITS-1	105
*max*-R	ACCCTTCTAAAGAGC		
*max*-probe	*VIC*-ATTAAGGACACAAACAATGCCTA-*MGB*		
*tene*-F	TTATGAGAGGAGAAGACG	*Eimeria tenella* AF446074 ITS-1	111
*tene*-R	AGACAGAACGCACACA		
*tene*-probe	*TXR*-ATGCAGAGCGCTCGCGGCTC-*MGB*		
*nec*-F	ACACAGTTTGTACGCCT	*Eimeria necatrix* AF446069 ITS-1	60
*nec*-R	AAGCTGACGCTTGAAAC		
*nec*-probe	*CY5*-AGAACGCGCTGCTGCTG-*MGB*		

**Table 2:** Primers used for recombinant plasmid standards construction.

Pathogens	Primers	Sequence (5′ → 3′)	Product Size (bp)	Accession No.
*Eimeria acervulina*	F	ACGACGCATTTTTGTG	214	AF446055
	R	GCTATGGGTGCTCATC		
*Eimeria* *maxima*	F	AGAACTAGCCTAACCC	138	AF446060
	R	ATGCAAGAGGACATC		
*Eimeria tenella*	F	GTGGAACCTCTCAAGA	431	AF446074
	R	TGATCCTGCGTTGTGA		
*Eimeria necatrix*	F	AGTAGAAGAGCCTATCA	292	AF446069
	R	TCATTCACACAGTTTGTAC		

**Table 3:** Intra-assay and inter-assay reproducibility of the multiplex TaqMan-MGB qPCR assay for standard plasmids p-acer, p-max, p-ten, and p-nec at three different template concentrations. Each plasmid standard was tested in triplicate across three independent experiments. The coefficient of variation (CV) was calculated based on the mean quantification cycle (Cq) values and standard deviations (SD).

Plasmid Standards	Concentration of Template (Copies/μL)	Intra-Coefficient of Variation		Inter-Coefficient of Variation	
		X ± SD	CV (%)	X ± SD	CV (%)
p-acer	10^7^	14.527 ± 0.270	1.86	14.680 ± 0.264	1.80
	10^6^	18.660 ± 0.235	1.26	18.630 ± 0.350	1.88
	10^5^	22.776 ± 0.287	1.26	22.753 ± 0.371	1.63
p-max	10^7^	13.79 ± 0.199	1.45	13.833 ± 0.220	1.59
	10^6^	18.567 ± 0.255	1.38	18.500 ± 0.266	1.22
	10^5^	23.247 ± 0.291	1.25	23.150 ± 0.260	1.13
p-ten	10^7^	21.547 ± 0.192	0.89	21.733 ± 0.380	1.75
	10^6^	25.573 ± 0.276	1.08	25.427 ± 0.387	1.52
	10^5^	29.607 ± 0.248	0.84	30.073 ± 0.318	1.06
p-nec	10^7^	14.257 ± 0.261	1.83	14.543 ± 0.215	1.48
	10^6^	17.523 ± 0.163	0.93	17.556 ± 0.261	1.49
	10^5^	22.827 ± 0.172	0.76	23.000 ± 0.209	0.91

## Data Availability

The original contributions presented in this study are included in the article. Further inquiries can be addressed to the corresponding author.
